# Cross-cultural evidence does not support universal acceleration of puberty in father-absent households

**DOI:** 10.1098/rstb.2018.0124

**Published:** 2019-02-25

**Authors:** Rebecca Sear, Paula Sheppard, David A. Coall

**Affiliations:** 1 Population Health, London School of Hygiene and Tropical Medicine, London, UK; 2 Department of Sociology, University of Oxford, Oxford UK; 3 Medical and Health Sciences, Edith Cowan University, Joondalup, Australia

**Keywords:** puberty, father absence, cross-cultural, menarche, family structure, early-life environment

## Abstract

Father absence in early life has been shown to be associated with accelerated reproductive development in girls. Evolutionary social scientists have proposed several adaptive hypotheses for this finding. Though there is variation in the detail of these hypotheses, they all assume that family environment in early life influences the development of life-history strategy, and, broadly, that early reproductive development is an adaptive response to father absence. Empirical evidence to support these hypotheses, however, has been derived from WEIRD (Western, Educated, Industrialized, Rich and Democratic) populations. Data from a much broader range of human societies are necessary in order to properly test adaptive hypotheses. Here, we review the empirical literature on father absence and puberty in both sexes, focusing on recent studies that have tested this association beyond the WEIRD world. We find that relationships between father absence and age at puberty are more varied in contexts beyond WEIRD societies, and when relationships beyond the father–daughter dyad are considered. This has implications for our understanding of how early-life environment is linked to life-history strategies, and for our understanding of pathways to adult health outcomes, given that early reproductive development may be linked to negative health outcomes in later life

This article is part of the theme issue ‘Developing differences: early-life effects and evolutionary medicine’.

## 1. Introduction

Puberty is a life-course transition of considerable interest to the evolutionary, social and health sciences. Health and policy-oriented disciplines have devoted much attention to it, given that, in higher income settings, early puberty has been shown to be a marker for adverse health outcomes in later life [1,2]. The evolutionary human sciences have been particularly interested in developing functional (i.e. evolutionary, ultimate) explanations about why some individuals should experience puberty earlier than others [3,4]. This latter literature has focused on the role of early-life experiences, particularly family environment, on the timing of puberty. A consensus that has emerged from this literature is that father absence in childhood is associated with younger age at menarche in girls [5,6]. Unusually, this is a consensus that has crossed over from the evolutionary to the non-evolutionary social sciences, as evidenced by the number of empirical studies that have tested this association by researchers not primarily motivated by an evolutionary theoretical framework [7]. Until recently, however, there was a significant gap in this literature: empirical research on this association was entirely conducted on WEIRD (Western, Educated, Industrialized, Rich and Democratic) populations [8]. This is problematic because WEIRD populations represent only a very narrow slice of humanity; in order to test evolutionary hypotheses, it is important to use data from a much broader range of human societies [8–10]. Recent research has begun to fill this gap by testing associations between father absence and age at puberty in non-WEIRD populations. Here, we review this new literature to assess the current state of knowledge on associations between family environment in early life and the timing of puberty, without relying exclusively on data from high-income populations.

## 2. A brief historical overview

In 1982, the anthropologists Draper & Harpending [11] wrote a seminal paper in the evolutionary human sciences which proposed that early-life environment should influence reproductive behaviour in later life, because early childhood involves a sensitive period of learning that determines children's developmental trajectories. In particular, they argued that: (i) father presence or absence in early childhood reflects the type of paternal investment that is typical in a particular environment; (ii) a different set of reproductive and behavioural strategies results in higher lifetime reproductive success in populations with relatively high, versus relatively low, levels of paternal investment; and (iii) children should use the cue of whether their father is present or absent to develop those reproductive and behavioural strategies that are best suited to high or low paternal investment, respectively. Though they acknowledge early in their paper that ‘father-absent’ societies include those in which there are relatively distant relationships between fathers and children, even though fathers may still be married to the child's mother, they assume throughout much of the paper that societies with low paternal investment are often characterized by unstable, short-term partnerships. The reproductive strategy that girls develop in father-absent societies then is one with early sexual activity—because there is no need to waste time on selective mate choice—and unstable relationships. By contrast, father-present girls develop a strategy of forming long-term, stable relationships; this involves delaying sexual activity, because they do invest time in searching for a partner who is willing and capable of high paternal investment. Barkow [12] subsequently suggested that a prediction from this hypothesis was that father-absent girls should experience earlier menarche.

Draper & Harpending's argument was intended to explain cross-cultural variation in life-history strategies, but subsequent research on early-life environment and reproductive development has shifted towards explaining individual-level variation. In 1991, Belsky *et al*. built on Draper & Harpending's work to propose that a stressful family context, including father absence, marital discord and job stresses, induces an adaptive psychosocial stress response and alters the child's attachment mechanisms, which leads to earlier reproductive development, a short-term mating strategy and low parental investment. This work not only generalized Draper & Harpending's argument beyond father presence or absence, but also proposed a proximate mechanism (attachment) through which early-life environment was linked to subsequent behaviour: this subsequently become known as the ‘psychosocial acceleration’ hypothesis [13].

Chisholm [14], in 1993, explicitly brought the framework of evolutionary life-history theory to bear on Belsky *et al*.'s hypothesis, and argued that parental absence and other childhood stressors were cues to high mortality risk. He drew on recent cross-species work which suggested that mortality risk was the key determinant of life-history variation: mammalian species that experience high mortality risk tend to mature early and give birth to many offspring; those that experience relatively low mortality mature late and have few offspring [15]. Chisholm argued that these cross-species observations could help explain within-species variation, and that children who experience cues to high mortality during early development should shift towards a reproductive strategy involving early maturation and high mating effort (many, unstable partnerships), whereas those who experience cues to low mortality should mature late and adopt a high parenting effort strategy (few children, with intensive investment in each).

A number of details in these arguments might be questioned. For example, the theoretical motivations for assuming that early maturation is necessarily associated with a short-term mating strategy of many, unstable partnerships appear weak (see the next section for more details). Further, in environments with low paternal investment, it could be argued that women should invest more in their own somatic capital and delay reproduction until they have achieved a larger body size (C. Moya 2013, personal communication). Nevertheless, Draper & Harpending's hypothesis and its subsequent developments have proved very influential in the evolutionary human sciences. These papers clearly laid out the hypothesis that environmentally induced shifts in development are adaptive responses to conditions experienced in early life, and spawned a large body of subsequent research motivated by exploring further whether, and how, early-life environment influences reproductive and behavioural outcomes in later life.

## 3. Subsequent development of Draper & Harpending's hypothesis

The hypotheses linking father absence in childhood with reproductive development have become more sophisticated, and have been added to, over time [3,4,16]. Empirical work on the psychosocial acceleration hypothesis has confirmed that it is a stressful family environment, rather than simple father absence or presence, that seems to have most power to explain early maturation [17–19]. Psychosocial stress from causes other than family relationships has also been shown to be associated with early puberty in high-income populations [20]; childhood sexual abuse, for example, has shown very consistent associations with earlier puberty [21]. Girls in WEIRD populations from socioeconomically disadvantaged families also experience earlier puberty than those from more advantaged backgrounds, which has been interpreted as further evidence that harsh early environments, with relatively high mortality rates, adaptively accelerate life-history strategy [22,23]. This led Ellis, in 2004 [4], to distinguish between Belsky *et al*.'s psychosocial acceleration hypothesis and the ‘paternal investment’ hypothesis, effectively a new name for Draper & Harpending's model. The paternal investment hypothesis maintains a special role of the father in determining reproductive development, and orienting girls towards a short-term, rather than long-term, mating strategy, but is also distinct from the psychosocial acceleration hypothesis in that it recognizes that father absence does not necessarily involve ‘stress’ in contexts where father absence is normative. Ellis and others reported support for independent associations between stressful life events, father absence and puberty in several empirical studies, suggesting father absence and psychosocial stress might be distinct axes of influence on child development [17,24,25].

In his 2004 paper, Ellis further developed the psychosocial acceleration and ‘paternal investment’ models into an additional ‘child development’ hypothesis [4]. This hypothesis proposes that reproductive development should be slowed down when high levels of parental, including biparental, investment are experienced, in order to capitalize on this investment and enter the reproductive arena only when a high level of individual capital (such as larger body size, higher skill or education level) is achieved. This argument assumes greater capital will lead to higher reproductive success, despite the delay to reproduction, in environments with high levels of parental investment [26]. A notable difference between this hypothesis and both psychosocial acceleration and ‘paternal investment’ hypotheses is that it focuses exclusively on the timing of maturation, and makes no predictions about how parental investment may influence mating strategies. This is an important point—that reproductive timing and mating strategies are distinct components of reproductive strategies—but one not always recognized in the human literature on early-life environment and reproductive development, where simplistic assumptions are sometimes made about how early maturation and short-term mating are inextricably linked as part of a ‘fast’ life-history strategy [27]. The assumption of a tight association between short-term mating and early maturation is absent from the non-human life-history literature, which focuses on the timing and number of reproductive events [15,28]. Although there may be trade-offs in the effort devoted to reproductive functions such as mating and parenting ([29] but see [30]), which may lead to some correlations between reproductive timing and mating behaviour, humans demonstrate plasticity in mating and parenting behaviour, so that there is likely to be some variation in exactly how these correlations play out [31]; there may also be sex differences in how these trade-offs are resolved.

Subsequent theoretical developments involved the proposal that, rather than early-life environment acting as a cue to future environments (an ‘external prediction’ model), as early work had assumed, early-life stress directly influences the individual's physiology and psychology, causing physiological changes that shift developmental trajectories (the ‘internal prediction’ model [32]. This is similar to the ‘weathering hypothesis' proposed by Geronimus to explain earlier childbearing in African-American mothers, compared with other groups in the USA, as a response to their more rapid deterioration of health resulting from the discrimination and disadvantage they face [33]). While the internal and external prediction models are not mutually exclusive, external prediction models do require extremely high levels of environmental stability between childhood and adulthood, which may perhaps be somewhat unlikely in a long-lived species such as our own [34]. Further, Matchock & Susman [35] have proposed a rather different hypothesis, suggesting that delayed reproductive development in the presence of the father is an inbreeding avoidance mechanism, whereas accelerated reproductive development when a stepfather is in the home is an adaptive response to the presence of an unrelated male. This is not a hypothesis that has received a great deal of attention, though it is possibly supported by a curious finding that menarche is later in girls who share a room with a father or brother than those who share a room with mother or sister in an Indian sample [36]. One final hypothesis that deserves mention is that father-absence associations may not be causal but simply reflect genetic confounding: families in which divorce and conflict are likely may also be those that have earlier puberty if these factors are genetically linked [37–39]. Research that has examined this possibility, however, has found that genetic confounding seems unlikely to entirely explain away associations between father absence and the timing of puberty [40–42].

There has therefore been considerable theoretical development of Draper & Harpending's hypothesis. Substantial empirical evidence has also emerged, to the point where Webster *et al*. [5] were able to conduct a meta-analysis in 2014 of 33 different analyses of the relationship between father absence and age at menarche. In these samples, the direction of the association was very consistent: their paper suggests that 32 of 33 found that father absence was associated with accelerated, as opposed to delayed, menarche. The authors' meta-analysis, including more than 70 000 participants, found a statistically significant association between father absence and accelerated menarche. Not acknowledged in this meta-analysis, however, was the homogeneity of these 33 samples. All were from WEIRD, and most were from WEEIRD (Western, English-speaking, Educated, Industrialized, Rich and Democratic), populations: 25 samples were from English-speaking populations (15 USA, 4 Australia, 3 New Zealand, 2 UK, 1 Canada; some estimated from authors’ affiliations because not all researchers stated the origin of their sample); only eight were from non-English-speaking populations (3 Poland, 2 Germany, 1 France, 1 Finland, 1 Bosnia and Herzegovina). This bias is potentially problematic because English-speaking WEIRD populations tend to have somewhat unusual reproductive patterns, even compared with other high-income populations. The USA, in particular, is a socioeconomically unequal population, where early childbearing is concentrated among disadvantaged socioeconomic groups [43]. The heavy weighting of this meta-analysis towards populations that show particularly unusual reproductive scheduling could potentially have an impact on the conclusions of this meta-analysis.

## 4. WEIRD populations are weird

An over-reliance on data from WEIRD populations, regardless of language spoken, is problematic, not only because such populations represent a very narrow slice of humanity, but also because such populations are rather weird in many respects, compared with most of humanity [8]. WEIRD populations are very different energetically, in that they have much greater access to food resources, have to expend considerably less energy to acquire those food resources, and expend less energy on immune defence. One consequence of this is that age at puberty is now several years earlier in WEIRD compared with non-WEIRD populations [44]. WEIRD populations are weird in terms of reproductive behaviour, particularly student populations, which were well represented among the samples included in the meta-analysis. These typically consist of large numbers of similarly aged young adults grouped together, away from the influence of parents or other family members, with female-biased sex ratios, and where short-term mating behaviours have relatively few consequences in terms of unintended pregnancy or sexually transmitted infections. WEIRD populations also have a rather weird family structure, in that the nuclear family, with an unusually extreme sexual division of labour [45], is considered the norm, with couples often living in geographical isolation from extended kin networks [46,47]. In such families, children are dependent on parents for much longer than is typically the case in human societies, and are expected to contribute little to the family economy. These family differences have implications for psychological theories of child development: attachment theory, for example, which forms an important component of the psychosocial acceleration hypothesis, has recently been criticized for cultural specificity [48,49]. The exclusively WEIRD focus of empirical work on family influences on reproductive development, as represented in Webster *et al.*'s [5] review, therefore raises questions about how generalizable these empirical results are.

## 5. A hierarchical model of father absence, acknowledging cross-cultural variation

Despite this empirical focus on WEIRD societies, Coall & Chisholm [50], in 2003, explicitly tackled the question of whether father absence and early-life stress would universally accelerate reproductive development. They argued for a hierarchical model, suggesting that accelerated development under conditions of psychosocial stress may only be possible, given a certain base level of resources, and that stress may instead *delay* development in less well-provisioned populations. This possibility was acknowledged by Belsky *et al*. [13], but the idea was subject to little theoretical development and few empirical tests in the intervening two decades. An exception was Waynforth's [51,52] testing of the psychosocial acceleration model in Ache hunter–gatherer and Mayan horticulturalist populations in South America. He did not have data on puberty but found that father absence (excluding cases due to paternal death) delayed first births in Ache women and Mayan men, though had no association with first births in Ache men; there was also some evidence that Mayan men who grew up in father-absent households were more oriented to a short-term mating strategy. These mixed results demonstrated the importance of testing hypotheses in non-WEIRD populations, as well as the importance of examining reproductive and mating outcomes separately, yet have been relatively rarely cited in the literature on father absence and reproductive development.

## 6. Anthropological research on paternal investment and adolescent outcomes

As evidenced by Draper & Harpending's interest in the topic, paternal investment has been of long-standing interest in anthropology, given that humans are a relatively unusual mammal in which fathers typically, though not universally, invest in their children [53]. The form paternal investment takes also varies between populations, and may include direct care and protection, provisioning with food or other resources, teaching, and social facilitation such as conferring social status, providing children with a kin group, and facilitating relationships with other social allies or mates [54,55]. Draper & Harpending's hypothesis about early-life family environment has been mainly developed in the evolutionary psychological literature; evolutionary anthropological research on paternal investment, instead, is typically concerned with more immediate impacts of paternal investment on child and adolescent outcomes. The assumption anthropological research starts from is that greater paternal investment, all else equal, will improve child outcomes and ultimately result in higher reproductive success. The simplest hypothesis about lack of paternal investment in childhood, therefore, is that this will *delay* children's development—assuming that early reproductive debut will increase lifetime reproductive success, which is a common finding, at least for women, across human populations [56]. This very simple explanation for the influence of paternal investment on the reproductive maturation of offspring has been tested, and appears to hold, in some other species that also have paternal care: yellow baboons [57], prairie voles [58] and male (but not female) marmosets [59].

While data on puberty are relatively scarce in the anthropological literature, several anthropologists have tested this simple model of paternal investment on behavioural measures of reproductive maturation such as the timing of first birth or first sex, and found that the absence of fathers is associated with delayed first births (in women: Gambia horticulturalists [60], Ache hunter–gatherers [52], pre-industrial Finns [61]; in men: Maya, Belize [51]; in both sexes: matrilineal Mosuo of southwest China [62]). This may be partly explained as a consequence of more rapid physiological development in children who receive paternal investment and provisioning in childhood, but may also be related to the roles fathers may play in launching their offspring into the reproductive arena, by helping arrange marriages or initiation ceremonies [63]. This simple model does not always hold, however statistically significant associations between father absence and age at first birth are not always seen (e.g. Ache men [52]; Dominican women [64]; some sub-Saharan African populations [65]; Tsimane forager-farmers [66]). Further, in several populations, earlier first births have been observed for father-absent offspring populations: an analysis incorporating 20 datasets from small-scale societies contributed by anthropologists found a consistent accelerating association between father absence and women's age at first birth, but not men's [67]. Finally, in an early (1988) paper on the subject, Flinn observed delayed entry into sexual and reproductive behaviour for father-present daughters in Dominica. He interpreted this finding as a consequence of fathers guarding daughters from the attentions of predatory men, a hypothesis that requires it to be adaptive for daughters to delay first births [68] and that aligns with research in the non-evolutionary social sciences showing that parental monitoring of adolescents is associated with delayed sexual and reproductive behaviour [69].

## 7. A gap in the evolutionary literature on father absence and reproductive development: the extended family

A feature of anthropological work on family environment that is notably lacking from the evolutionary literature on early-life family structure and reproductive development is the influence of family members beyond the father. It is acknowledged in the psychosocial acceleration model that it may be beneficial for women to reproduce early in low paternal investment environments because young women can benefit from the help of their mothers and other older female kin [13]; even in WEIRD societies, evidence suggests grandparental investment may replace paternal investment for young mothers [70]. But otherwise, little attention is paid to the wider family environment in this literature. Evolutionary anthropological research suggests that humans are cooperative breeders (loosely defined as requiring help from individuals other than the mother in childrearing), using a relatively flexible strategy in that help comes from a range of different sources, including fathers but also grandmothers, older siblings and men other than the child's father [71,72]. One implication, for some early-life hypotheses, is that paternal absence may have different consequences according to who else is available to help mothers raise children. Hypotheses that rely on family disruption or stress causing downstream effects on reproductive development may have little predictive power in societies where paternal investment is easily substitutable by individuals other than the child's father, such as partible paternity societies in South America, or where grandmothers, rather than fathers, have particularly important roles in the lives of young children.

Evolutionary anthropologists researching the cooperative breeding hypothesis have shown that father absence seems to have surprisingly few associations with child outcomes, such as survival in early life, possibly because paternal investment can be substituted by investment from other individuals in some populations [73,74], whereas the presence of grandmothers tends to be more strongly associated with child survival. This begs the question of why father absence in early life—while seemingly often unimportant in terms of contemporaneous child outcomes—should nevertheless have an impact on shaping the developmental trajectories of their offspring? Is this because research on paternal influences on child mortality and on reproductive development has typically been conducted in non-WEIRD and WEIRD populations, respectively, and paternal influences are rather different in the two types of society?

One new hypothesis that has been proposed to explain why parents should influence their offspring's reproductive development does relate to the idea that humans are cooperative breeders: this is the hypothesis that age at first birth is influenced by intergenerational conflict ([75] see also [76]). In cooperative family systems, there may be conflict over who gets to reproduce within a family: parent or adolescent offspring. Asymmetries in genetic relatedness suggest that parents will often ‘win’ these conflicts: parents will have more success at persuading adolescent children to help raise their siblings (because siblings are related at *r* = 0.5) than the adolescent children will have at persuading their parents to help raise the adolescent's own offspring (who are their parents' grandchildren, and so related at *r* = 0.25). This will delay the offspring's age at first birth, but only in households where additional children born into the household are full siblings of the adolescent children. Where the adolescent's father is no longer present in the household, she will have less incentive to stay in the natal home and help rear half-siblings, and she will accelerate her reproductive development.

The complexity of human societies, and the varied roles that fathers, and other family members, play in the lives of their offspring, means that there is unlikely to be a single, simple explanation for associations between father absence and reproductive development. Multiple hypotheses may need to be considered when interpreting paternal influences on offspring reproductive development (see table 1 for a list of these hypotheses), and it should be acknowledged that paternal influences may vary between populations. Anthropological research also highlights that, while there may well be an influence of early-life family environment shaping reproductive development, the continued role fathers play throughout their children's lives, even into young adulthood, means that the presence or absence of fathers should also be considered at later developmental stages. This is particularly important for behavioural life-history outcomes such as the timing of first sex and first birth, but the physiological process of puberty also occurs over many years, and may continue to be subject to paternal investment in later childhood or early adolescence, especially in populations where puberty occurs relatively late. Finally, evolutionary anthropological research also raises the question: what is the impact of the absence of other important carers, such as grandmothers, in early life; does grandmother absence also accelerate reproductive development?

**Table 1.** List of hypotheses that predict an association between father absence (FA) in childhood and the timing of puberty.

Predictions/Hypothesis	Association with father absence	Sensitive period in early childhood?	Paternal death or parental divorce important?	Maternal absence accelerates puberty?	Applies to boys, girls or both?	Predicts early childbearing?	Early maturation and short-term mating are linked?	Cross-culturally universal?*	Refs
‘Paternal investment': FA cue to paternal investment in environment	accelerating	yes	divorce	no	girls	yes	yes	yes?	Draper & Harpending [11]; Barkow [12]
Father presence allows 'daughter-guarding'	accelerating	no	both	maybe	girls	yes	yes?	no?	Flinn [68]
‘Psychosocial acceleration': FA alters attachment mechanisms and shifts development towards earlier maturation	accelerating	yes	both	yes	both	yes	yes	yes?	Belsky *et al.* [13]
FA cue to mortality risk in the environment	accelerating	yes	both	yes	both	yes	yes	yes?	Chisholm [14]
FA indicates lack of paternal investment, which slows down physiological development and entry to marriage market	delaying	no	both	yes	both	yes	no	no?	Winking *et al.* [66]; Scelza [63]
Child development': Father presence reflects high parental investment, so maturation should be delayed to capitalise on this	accelerating	no	both	yes	both	yes	no	yes?	Ellis [4]
Father presence reflects the potential for inbreeding for daughters	accelerating	no	both	no	girls	yes	no	yes?	Matchock & Susman [35]
‘Internal prediction': FA adversely affects internal state which shifts development towards early reproduction	accelerating	no	both	yes	both	yes	no	no?	Rickard *et al*. [32]
FA reflects lack of intergenerational conflict	accelerating	no	both	yes	both	yes	no	no?	Moya & Sear [75]
FA reflects genetic correlation of earlier maturation and short-term mating strategies	accelerating	no	divorce	no	girls	yes	yes	yes?	Comings *et al*. [39]; Barbaro *et al*. [38]

We have tried to generate predictions based on our reading of the original formulation of each hypothesis (rather than subsequent developments or our own opinions about what these hypotheses should predict).

*By 'Cross-culturally universal' we mean 'if fathers were absent, would this hypothesis always predict accelerated/delayed maturation to follow (all else - including resource access - equal)?’

For columns marked 'Yes?', some hypotheses acknowledge that other factors (such as low resource access) would override father absence, but still fundamentally assume that the mechanism underlying the FA and puberty relationship is universal.

For columns marked 'No?', most hypotheses assume that variation in paternal investment or family relationships would lead to variation in the FA-puberty association cross-culturally.

Our own opinion is that - given paternal investment and family relationships do vary cross-culturally - it is unlikely that any of these hypotheses will apply cross-culturally.

## 8. What do the data show when non-WEIRD populations are included?

Here, we update Webster *et al*.'s 2014 [5] literature search, and present information from a number of new studies, many from non-WEIRD contexts, which have investigated the association between father absence and age at puberty. We include studies on associations between father absence and the timing of puberty in boys. Most of these are included in Xu *et al*.’s [77] 2018 meta-analysis of early-life environment and reproductive outcomes in boys, but we have updated their review with an additional three studies. We include only studies published in English in our review, and exclude data from unpublished dissertations or conference presentations. We do not perform any meta-analyses on these data. We consider a meta-analysis is inappropriate because the studies vary considerably in quality. Some only present univariate analysis, others control for multiple confounders, sometimes including maternal age at menarche, a potential control for genetic confounding. The sampling strategies also vary considerably, ranging from large nationally representative surveys to small convenience samples. The studies also vary in the operationalization of both puberty and father-absence variables, and several studies run tests on different versions of both variables, sometimes finding different results for different operationalizations. Finally, not all datapoints are independent, with some countries, and even some datasets, represented multiple times. Any formal meta-analysis of these data may therefore give false confidence to the conclusions we draw here. Further, our *a priori* theoretical prediction is *not* that father absence will be universally associated with early puberty across all populations: we are more interested in a data-driven approach to understanding how associations between father absence and the timing of puberty may vary between populations.

**Figure 1.**
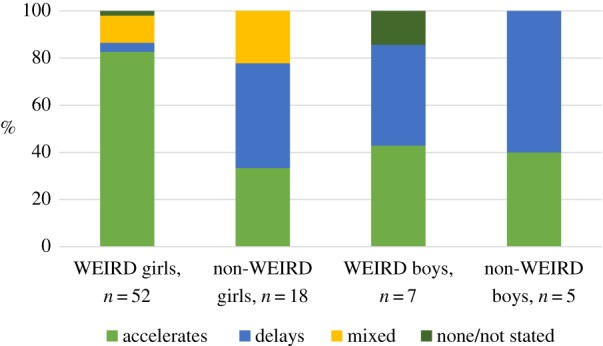
Percentage of studies that found accelerating, delaying, mixed or no associations between father absence and the timing of puberty, regardless of statistical significance. (Online version in colour.)

**Figure 2.**
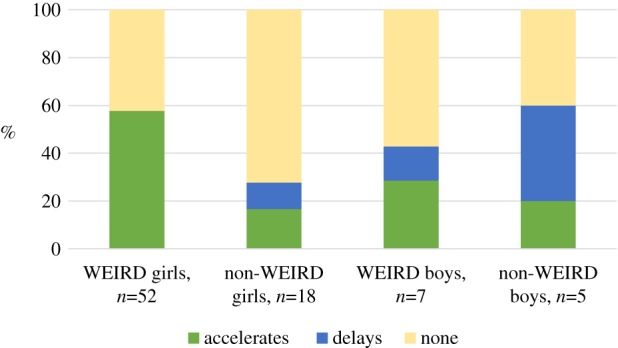
Percentage of studies that found significantly accelerating, delaying or non-significant associations between father absence and the timing of puberty. (Online version in colour.)

Figure 1 summarizes the data from all studies we have found investigating associations between father absence and the timing of puberty (a full list of studies can be found in the electronic supplementary material, table S1). We separate out data from WEIRD and non-WEIRD societies for illustrative purposes, though we acknowledge the very considerable diversity not only among non-WEIRD societies (which represent the vast majority of human populations over time and space) but also within WEIRD populations. The figure shows the percentage of studies that have found accelerated, delayed, mixed or no associations between father absence and the timing of puberty for girls and boys. ‘Mixed’ means that associations were both accelerated and delayed depending on the operationalization of the father absence (e.g. [78]) or puberty variable (e.g. [79]); ‘none’ that the correlation was zero. We have coded each study according to the direction of the association found, regardless of statistical significance. Figure 2 shows the same data but with only statistically significant studies in the ‘accelerated’ or ‘delayed’ groups, and non-significant results classified as ‘none’.

Figure 1 confirms that WEIRD girls show consistent associations between father absence in childhood and earlier puberty (*n* = 52 studies, mean sample size = 2578, range 71–21 437). There are far fewer studies on girls from non-WEIRD populations (*n* = 18, mean sample size = 1385, range 87–11 138; including two WEIRD-ish populations—from current high-income populations, but not contemporary data), and on boys (WEIRD *n* = 7, mean sample size = 2357, range 78–9596; non-WEIRD *n* = 5, mean sample size = 1333, range 206–4749; including one WEIRD-ish), but these studies show much more variation, with associations between father absence and both accelerated and delayed puberty observed. Our substantive conclusions are the same if we take statistical significance into account. Figure 2 shows that studies on WEIRD girls are relatively consistent, in that a majority—approximately 60%—show significant accelerating associations between father absence and the timing of puberty. Studies on boys or non-WEIRD girls are more variable: fewer studies on non-WEIRD girls and WEIRD boys show significant associations; although around 60% of studies on non-WEIRD boys are significant, these associations are split between delaying and accelerating associations. Because our substantive conclusions are similar regardless of statistical significance, and to avoid giving too much emphasis to statistical analysis from studies of varying quality, the next few sections discuss results ignoring statistical significance (unless otherwise stated).

Examining the new studies we have found for girls (i.e. those not included in the Webster *et al*.'s 2014 review [5]), we find considerable variation both in the timing of puberty and in family structure (see details in electronic supplementary material, table S2). Where menarche was measured, it varies from an average age of around 11 to 14 years. Regarding family environment, there was considerable diversity in the percentage of girls living without their fathers: ranging from 2% in Nepal to more than half in Uganda. Such cross-cultural variation will prove useful in future studies attempting cross-population analysis to investigate under which circumstances father absence is associated with reproductive development. We have also included information on whether the timing of, and reason for, father absence, and mother absence, was analysed in each study, as such data may allow us to draw some conclusions about which hypotheses linking father absence to the timing of puberty receive most support (see table 1). We comment on these factors below, though our overall conclusion is that not enough studies have examined them in sufficient detail to allow any firm conclusions to be drawn.

## 9. Timing of father absence

Information on when father absence in childhood occurred is useful, given that hypotheses relating early-life family environment to maturation typically assume there is a sensitive period in early childhood during which family environment shapes maturation; whereas hypotheses that relate to the direct impact of fathers on maturation do not rely on sensitive periods to the same extent. Investigating the timing of father absence may therefore both allow determination of whether early-life hypotheses are being tested in the most rigorous way, and potentially distinguish between alternative hypotheses for paternal influence on reproductive development. The studies included in the electronic supplementary material, table S2 show some variation in the timing of father absence; some are only able to assess father absence relatively late in childhood (e.g. in Indonesia), with several using family structure at the time of data collection as the predictor variable, rather than father absence in early life; others assess only early father absence; while a few are able to test whether father absence in early and late childhood is associated with maturation (e.g. Malaysia, Kinsey, Curaçao). While there did appear to be a tendency for analyses that tested father absence in early childhood to be associated with accelerated, rather than delayed, puberty (Malaysia, Kinsey survey, Maya, South African whites and blacks), this was not universally the case (South African mixed race, UK school sample). Moreover, those studies that are able to test whether the results differed according to early versus late absence found mixed results: in Malaysia, early absence is associated with accelerated puberty, late absence with delayed; in the US Kinsey survey, results differed according to whether father absence was due to death or divorce; and in Curaçao, absence in both early and late childhood is associated with accelerated puberty.

## 10. Death versus divorce

There may also be differences in the implications of father absence for children according to why fathers are absent (e.g. [80,81]). Hypotheses that rely on father absence as a cue to future mating environments imply that only father absence through parental divorce, not paternal death, may show associations with children's maturation. Hypotheses that cite psychosocial stress as the primary mechanism in accelerating development may expect associations between family instability for any reason and earlier puberty. A few studies in the electronic supplementary material, table S2 have distinguished between father absence due to death or divorce, but with similarly inconclusive results. For example, the two studies we (P.S., R.S.) have conducted find opposite associations with puberty depending on whether divorce or paternal death is the predictor variable: in Malaysia, paternal death is associated with delayed puberty, divorce with accelerated puberty; in the US Kinsey survey, death is associated with accelerated, and divorce delayed, puberty [82,83]).

## 11. Mother versus father absence?

Different hypotheses may predict different associations between mother absence and puberty: those relying on father absence as a cue to mating environment make no predictions about mother absence, whereas mother absence is certainly likely to cause psychosocial stress [22]. We find that there are associations in both directions (delaying in the Philippines and China; accelerating in Iran; with mixed associations depending on the timing of absence in the US Kinsey survey), but mother absence rarely seems to be significantly associated with the timing of puberty, possibly because of the small sample sizes of mother-absent children (but see [84] for an early cross-cultural examination).

## 12. Updating the literature on WEIRD girls

For WEIRD populations, our updated literature search supports Webster *et al*.'s conclusion [5] that WEIRD populations show more consistency in the direction of the association between father absence and the timing of puberty. It is worth noting, however, that the additional studies we found slightly increase the proportion of studies that observe delaying associations between father absence and puberty, and that observe no statistically significant associations. Also now included in the WEIRD list are results from several studies that repeat similar analyses on the same datasets (such as the US Add Health dataset, and the UK Avon Longitudinal Study of Parents and Children (ALSPAC dataset), and these repeated analyses often find different results. This may be partly explained by heterogeneity in the exact sample chosen. For example, when analysing Add Health data from the USA, different results are observed when only including white participants [40] versus all ethnic groups [85]. But this, along with the increasing number of studies that find delaying or non-significant associations, may also suggest that the fundamental correlation between father absence and puberty is not necessarily robust to alternative model specifications [86]. More recent studies tend to use more sophisticated analysis than early studies–for example, including a wider range of additional variables in the model, such as variables that may provide better indicators of familial stress than father absence alone. For example, when the US Add Health longitudinal database is analysed, early father absence is significantly associated with earlier menarche [85] until a variable for parental emotional harshness is included in the model [21]. One study also explicitly tests an alternative hypothesis to father absence accelerating puberty: Smith [87] finds support for the intergenerational conflict model, because he finds that the association between father absence and age at menarche loses significance once the number of half- and step-siblings is included in the model. These new studies therefore perhaps suggest that, even in WEIRD societies, simple father presence or absence may not be very consistently associated with accelerated puberty. The apparent universality in early WEIRD results may also indicate a bias in earlier studies towards publication of analyses that found support for the father-absence hypothesis: for example, in an early review of the literature in 1997, Kim *et al*. [88] report non-significant associations between father absence and menarche in three conference presentations from studies that do not appear to have been subsequently published.

## 13. What about boys?

Boys have been somewhat neglected in the literature on early-life stress and reproductive development (but see [89]). Theoretical reasons are occasionally offered for this, though this is also likely driven by the relative lack of data on puberty for boys. It has been suggested that the trade-off between growth and reproduction is more important in determining female, compared with male, reproductive success, so the early-life environment is more likely to affect the timing of maturation in girls than boys [4]. Alternatively, it has been proposed that girls are more sensitive to the social environment than boys [90], or that female reproductive physiology should be more sensitive to environmental conditions generally, given their typically greater female parental investment [91]. These latter arguments, however, are in contrast with the general finding that boys' health is more sensitive to environmental condition than girls’ [92].

Examining the 12 studies that have been conducted on boys (7 WEIRD and 5 non-WEIRD, including 1 WEIRD-ish) in more detail (see electronic supplementary material, table S3), we see variable associations between father absence and the timing of puberty, with delaying and accelerating associations observed in both WEIRD and non-WEIRD samples. Only half of the studies find statistically significant associations, and these are evenly split between WEIRD and non-WEIRD samples, and between accelerating and delaying associations. Of those studies that investigate associations for both boys and girls, they do not always find the same association (e.g. early father death is associated with accelerated puberty for girls but delayed puberty for boys in the US Kinsey survey). Opposite associations between early-life stress and the timing of puberty for male and female offspring have also been observed in one study of rats exposed to early-life stress [93]. These findings suggest that males and females may not respond in the same way to early-life experiences.

## 14. Discussion

In WEIRD populations, where energy availability is high, mortality rates are low, reproductive development is early, and where a normative nuclear family is emphasized, father absence seems to be relatively consistently associated with earlier puberty in girls, but not boys. Outside of this narrow slice of humanity, however, associations between father absence and the timing of puberty are much more variable for both sexes; delaying and accelerating associations are found, although most associations are not statistically significant. This fits, to some extent, with the hierarchical model of father absence that accelerating effects of early stress will only be seen in relatively well-nourished populations [50]. This survey provides less support for the hypothesis that there is something universally special about fathers, such as being a cue to later life mating environment, that shapes children's life-history strategy in a particular direction (see also [94], which finds that menarche is earlier in girls who grow up in monogamous, versus polygynous, families, in contrast with Draper & Harpending's prediction). These findings are also consistent with the idea that fathers have multiple influences on their offspring, including perhaps both early-life effects shaping reproductive strategies and also more direct paternal care influences throughout development, at least some of which vary between populations.

It might be argued that the comparison of WEIRD and non-WEIRD populations is potentially problematic because of systematic differences between WEIRD and non-WEIRD analyses. Some of the non-WEIRD studies included, for example, present simple univariate associations and are based on biased samples of girls not all of whom have experienced menarche. However, many of the WEIRD studies also come from convenience samples (see Sohn [95] for more detail on the problematic nature of many of the WEIRD samples). Further limitations to comparability are that the timing of, and reason for, father absence may differ between WEIRD and non-WEIRD populations: father absence is more likely to be due to paternal death in non-WEIRD than WEIRD populations, given high mortality rates, for example. But several of the WEIRD studies have been able to exclude paternal death, or separate paternal death from divorce, and results still differ from the consistent accelerating association seen in WEIRD populations. A more significant problem may be that the empirical literature in WEIRD societies has developed beyond analysing simple father absence or presence to investigate in much more detail exactly what features of the family environment are most strongly associated with reproductive development. As yet, there is little research in non-WEIRD populations that has attempted to explore the family environment beyond relatively simple indicators of family structure. We also note that we define WEIRD societies rather conservatively, by classifying studies on current high-income populations that do not use contemporary data as non-WEIRD. Reclassifying all populations with majority European ancestry as WEIRD would not change our substantive conclusions, however. Though this would introduce a little more variation into the WEIRD data, WEIRD studies would still show consistently more accelerating associations than non-WEIRD studies.

## 15. Recommendations for future research

We have not yet completed a systematic review of the literature on the timing of other reproductive developmental outcomes, such as age at marriage or first birth, but several of the papers included in our review also investigate associations between father absence and markers of reproductive development other than puberty. A number of these studies find no statistically significant associations between father absence and puberty, but do find significant associations with outcomes such as age at first birth (e.g. father-absent girls in Malaysia have earlier first births though not earlier menarche [65]; and father-absent boys in the UK have earlier first births despite delayed puberty [96]). Firm conclusions must await a more thorough review of the literature, but these findings hint that family disruption in childhood may be more consistently associated with accelerated behavioural reproductive outcomes (such as age at first sex or first birth) than the physiological outcome of accelerated puberty (while noting the caveat that our earlier brief review on the anthropological literature suggests that paternal influences on even behavioural reproductive outcomes can be cross-culturally variable). What this potential disjunct between physiological and behavioural maturation does suggest is that it may be too simplistic to argue that father absence, or other early-life conditions, kickstarts a coordinated suite of life-history characteristics, involving puberty, reproductive and mating outcomes (often now referred to in the literature as ‘fast’ or ‘slow’ life-history strategies). While it is theoretically plausible that life-history characteristics, including mating and reproductive behaviour, hang together in consistent ways, there has been relatively little empirical testing of this assumption, and the empirical research that does exist has offered mixed results [97,98] (see also [99] for a theoretical critique). We recommend that future research adopts a data-driven approach of testing whether father absence is associated with multiple different outcomes, physiological and behavioural, and reproductive and mating, and evaluating whether and how the timing of, reason for, and sex of, parental absence are associated with these outcomes. Such a data-driven approach will help tease apart which hypotheses have most power in explaining any associations observed in a particularly context.

In order to progress our understanding of early-life influences on reproductive development, we further recommend greater integration of the various literatures that have contributed to this discussion. Much of the literature on early-life influences on reproductive development has been produced by disciplines that tend to focus exclusively on WEIRD populations in the social sciences, such as psychology and sociology. These disciplines have produced valuable data, useful methods and detailed analyses on the psychosocial mechanisms that mediate (but not moderate) such effects. Other disciplines have produced much relevant work on both reproductive development and the family environment, such as anthropology, biology and the health sciences, including the global health literature. There is a large literature, for example, in human biology and the health sciences, which has also been interested in how early-life environment affects later health and reproductive outcomes, but which has focused on nutritional or physiological insults in early life, demonstrating that lack of energetic resources is consistently associated with delayed maturation [100,101]. To really understand a process such as puberty, which is a physiological process but clearly influenced by the psychosocial environment, it would be helpful to bring together the diverse social and biological literatures on reproductive development.

Greater integration between the health, energetics and social science literatures would allow a much more detailed consideration of the mechanisms by which the early-life environment influences reproductive development. For example, in populations with lower resource access, a closer examination of the separate roles of low resource access and family instability and their interaction will be possible (low resource access and family instability may perhaps map onto environmental harshness and unpredictability, which are predicted to have independent influences on life-history strategy: [102]). This integration may also include bringing into the study of early-life environment an investigation of the prenatal period [22,103], and of maternal effects, including intergenerational effects [104]: in mammals, especially those whose period of childhood dependency is relatively long, as in humans, the mother has a significant influence on the development of offspring [105]. While the mother's parenting style has received considerable attention in the father-absence literature, her physiology has not.

Greater integration with the non-human literature may also bring benefits. For example, while our results do fit with a hierarchical model of father absence, they also raise the question: if father absence only consistently accelerates puberty in populations with a level of resource access that has only recently been seen in our species, is it really the result of an evolved, adaptive response? Investigation of the non-human literature may help here. If we find evidence that, under some circumstances, stress may accelerate maturation in other species, this may bolster the hierarchical hypothesis. Hrdy suggests the opposite tends to be the case, however: stressed and low-ranking primate females delay, rather than accelerate, menarche ([106], e.g. [107]). Low levels of paternal care have also been shown to delay maturation in male marmosets, in comparison with higher levels of paternal care [59]. But there are some studies which suggest that stress and psychosocial adversity in early life may accelerate reproductive development in non-human species [108]: female rats accelerate reproduction—though male rats delay—with disrupted maternal investment [93]; and in female marmosets, lower levels of paternal care accelerate maturation.

Greater integration with the non-human literature might help clarify the assumptions that are built into the hypotheses presented in table 1, improving the theoretical underpinnings of the literature on father absence and development. For example, dispersal is a key characteristic that influences family relationships and relatedness, and has received considerable attention in the non-human literature [109,110]. Yet, the human literature on father absence and development is largely silent on whether dispersal patterns matter for the hypotheses proposed, beyond what appears to be an implicit assumption that offspring remain in the same population as parents. Formalizing verbal arguments would also help clarify the assumptions built into the hypotheses proposed. As has been recently noted, the life-history literature broadly suffers from a lack of formal mathematical modelling [111,112], and the father-absence and development literature is no exception (but see [75]). Improving the theoretical basis for the verbal arguments that have been proposed would help advance the field.

## 16. Cross-cultural research allows testing of hypotheses about variation in early-life effects

Finally, we recommend greater integration between the literatures which have investigated father absence in WEIRD societies and the anthropological literature on the family; we further recommend expanding the number of studies which investigate the relationship between father absence and puberty in non-WEIRD populations. Opening up research to a broader range of societies allows the possibility not just for testing whether particular versions of the father-absence hypothesis receive support across a range of societies, but also for testing hypotheses about why paternal effects on later life outcomes should vary between societies. Given that societies differ in the role of fathers, and of other family members, it is not surprising that—when individual-level variation is considered—paternal effects vary between societies. These ideas about variation between populations are often acknowledged in the theoretical literature on father-absence effects; indeed, variation in paternal investment is precisely what kicked off this entire literature. However, the early switch in this literature from explaining between-population variation in reproductive development to explaining individual-level variation means that between-population differences have been neglected, and that the empirical literature in this area often gives the impression that father absence should be universally associated with early puberty.

Some versions of the hypothesis, which have been developed in the evolutionary developmental literature and tested in WEIRD populations, may work in WEIRD populations but perhaps not in populations with different family norms and structures. The psychosocial acceleration argument, for example, seems to imply that intensive paternal investment in early childhood is normative, and its absence causes children stress. It is plausible that this hypothesis works in societies that emphasize the nuclear family form, because the absence of a father may be considered socially problematic, as well as resulting in a significant loss of social networks and resources to the household. But, it may not hold such predictive power in societies in which paternal investment is more easily substituted by other individuals, such as where grandmothers and siblings have important caring roles for young children, or in ‘partible paternity’ societies in south America (this distinction between ‘contra-normative’ and ‘normative’ father-absent societies was highlighted in Draper & Harpending's original article).

Ellis’ child development model [4] may also have more predictive power in higher income, than lower income, populations. This model assumes that investing in embodied capital will bring benefits in later life, including successful reproduction, which may be particularly relevant for low fertility, post-demographic transition societies where investment in capital, such as educational capital, is important for success [26]. Conversely, the intergenerational conflict model may have more predictive power where offspring make significant contributions to the parent's household, i.e. outside WEIRD contexts. We are now getting to the stage where variation in paternal effects on reproductive timing can be investigated cross-culturally, to evaluate different hypotheses (e.g. [67]).

## 17. Conclusion

While the large body of work on early-life family environment and reproductive development originated in a hypothesis proposed by anthropologists, this literature has strayed away from its anthropological roots by focusing very largely on WEIRD populations. The results of the review presented here suggest that limiting environmental variation by restricting empirical research to such a narrow slice of humanity may distort the conclusions of this literature: associations between one particular aspect of early-life environment and reproductive development—father absence and the timing of puberty—look quite different when contexts beyond WEIRD populations are considered. These differences may be relatively easily incorporated into the theoretical frameworks used in this literature, but they also suggest that these theoretical frameworks may need closer examination, and certainly require more detailed testing across a broader range of human societies. Our opinion is that the variation in family organization and paternal investment seen across human populations means that associations between father absence and the timing of puberty are likely to vary between populations; future research needs to focus on developing theoretical frameworks and producing empirical evidence to explain how and why associations between early life experiences and reproductive development vary between populations.

## Supplementary Material

Supplementary Table 1

## Data accessibility

All data used are available in the electronic supplementary material.
